# Comparative metabolism of aflatoxin B_1_ in mouse, rat and human primary hepatocytes using HPLC–MS/MS

**DOI:** 10.1007/s00204-023-03607-z

**Published:** 2023-10-05

**Authors:** Andrea Gerdemann, Benedikt Cramer, Gisela H. Degen, Jannik Veerkamp, Georgia Günther, Wiebke Albrecht, Matthias Behrens, Melanie Esselen, Ahmed Ghallab, Jan G. Hengstler, Hans-Ulrich Humpf

**Affiliations:** 1 Institute of Food Chemistry, University of Münster, Corrensstraße 45, 48149 Münster, Germany; 2 Leibniz Research Centre for Working Environment and Human Factors (IfADo), Ardeystraße 67, 44139 Dortmund, Germany; 3 Department of Forensic Medicine and Toxicology, Faculty of Veterinary Medicine, South Valley University, Qena, 83523 Egypt

**Keywords:** Mycotoxins, DNA adducts, Protein adducts, Species differences, HPLC–MS/MS, Biotransformation

## Abstract

Aflatoxin B_1_ (AFB_1_) is a highly hepatotoxic and carcinogenic mycotoxin produced by *Aspergillus* species. The compound is mainly metabolized in the liver and its metabolism varies between species. The present study quantified relevant AFB_1_- metabolites formed by mouse, rat, and human primary hepatocytes after treatment with 1 µM and 10 µM AFB_1_. The use of liquid chromatographic separation coupled with tandem mass spectrometric detection enabled the selective and sensitive determination of phase I and phase II metabolites of AFB_1_ over incubation times of up to 24 h. The binding of AFB_1_ to macromolecules was also considered. The fastest metabolism of AFB_1_ was observed in mouse hepatocytes which formed aflatoxin P_1_ as a major metabolite and also its glucuronidated form, while AFP_1_ occurred only in traces in the other species. Aflatoxin M_1_ was formed in all species and was, together with aflatoxin Q_1_ and aflatoxicol, the main metabolite in human cells. Effective epoxidation led to high amounts of DNA adducts already 30 min post-treatment, especially in rat hepatocytes. Lower levels of DNA adducts and fast DNA repair were found in mouse hepatocytes. Also, protein adducts arising from reactive intermediates were formed rapidly in all three species. Detoxification via glutathione conjugation and subsequent formation of the *N*-acetylcysteine derivative appeared to be similar in mice and in rats and strongly differed from human hepatocytes which did not form these metabolites at all. The use of qualitative reference material of a multitude of metabolites and the comparison of hepatocyte metabolism in three species using advanced methods enabled considerations on toxification and detoxification mechanisms of AFB_1_. In addition to glutathione conjugation, phase I metabolism is strongly involved in the detoxification of AFB_1_.

**Supplementary Information:**

The online version contains supplementary material available at 10.1007/s00204-023-03607-z.

## Introduction

**Fig. 1:**
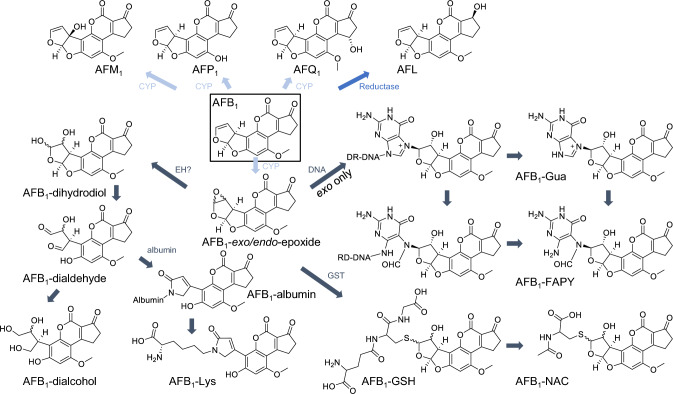
Phase I metabolism of AFB_1_ to AFM_1_, AFP_1_, AFQ_1_, and AFL. AFB_1_-epoxide binds to macromolecules such as proteins and DNA. Their downstream products AFB_1_-Lys and AFB_1_-Gua are used as long- and short-term biomarkers for AFB_1_ exposure. Efficient detoxification via glutathione conjugation reduces adduct formation (*EH* epoxide hydrolase, *GST* glutathione-*S*-transferase, *CYP* cytochrome P450)

Aflatoxin B_1_ (AFB_1_) is a well-known secondary metabolite mainly produced by *Aspergillus flavus* and *Aspergillus parasiticus* (Dohnal et al. 2014). These fungal species grow on various crops, especially in warm regions with high humidity, which are therefore highly endangered for aflatoxin contamination in food and feed (Rushing and Selim 2019). The compound is highly toxic and carcinogenic related to a mutagenic mode of action (Kensler et al. 2011) and toxicological properties, summarized in a recent opinion from the European Food Safety Authority (EFSA CONTAM PANEL 2020). The metabolism of AFB_1_ varies among species, which is known from early in vitro experiments using microsomes (Moss et al. 1985; Ramsdell and Eaton 1990; Yourtee et al. 1987; Roebuck and Wogan 1977) and studies reviewed elsewhere (Dohnal et al. 2014; Rushing and Selim 2019). As depicted in Fig. 1, AFB_1_ is metabolized by cytochrome P450 enzymes (CYP), which catalyze demethylation to aflatoxin P_1_ (AFP_1_), hydroxylation to aflatoxin M_1_ (AFM_1_) and aflatoxin Q_1_ (AFQ_1_) or epoxidation of the compound (Deng et al. 2018; Ramsdell and Eaton 1990). Also, the formation of aflatoxicol (AFL) upon reduction of AFB_1_ is a well-known metabolic step (Wong et al. 1979). Most phase I metabolites such as AFP_1_, AFM_1_, AFQ_1_, and AFL exert lower cytotoxicity than AFB_1_ (Buchi et al. 1973; Green et al. 1982; Neal et al. 1998; Wu et al. 2021; Yourtee et al. 1987). In contrast, the *exo*-epoxide is a highly reactive intermediate which binds covalently to the guanine residue of DNA (van Vleet et al. 2002; Hanigan and Laishes 1984). This bioactivation reaction is considered critical for the mutagenicity and carcinogenicity of AFB_1_ (Eaton and Gallagher 1994; Essigmann et al. 1977). The AFB_1_-guanine adduct can be converted to a rather persistent formamidopyrimidine (FAPY) adduct which is mainly responsible for the mutagenicity of AFB_1_ since the guanine adduct is repaired more efficiently (Smela et al. 2002).

The main mechanism for detoxification of the epoxide is the conjugation with glutathione (GSH) to an AFB_1_-GSH adduct which is further metabolized to the *N*-acetylcysteine (NAC) derivative (Moss et al. 1985; Degen and Neumann 1978; Raj and Lotlikar 1984). The toxicity of AFB_1_ in various species is therefore related to the efficiency of this detoxifying conjugation reaction (Murcia and Diaz 2021; Degen and Neumann 1981; Ilic et al. 2010). Furthermore, the epoxide is rapidly hydrolyzed to AFB_1_-dihydrodiol which is in equilibrium with the reactive AFB_1_-dialdehyde (Neal and Colley 1979). To what extent this hydrolysis is catalyzed by epoxide hydrolases or if it is spontaneously generated due to the instability of the compound is under discussion (Guengerich et al. 1998; Johnson et al. 1997). Nevertheless, the formed dialdehyde can further react with primary amines such as the ε-amino group of lysine present in serum albumin, which makes the determination of AFB_1_-lysine (AFB_1_-Lys) adducts in blood samples a reliable long-term biomarker for AFB_1_ exposure (Sabbioni et al. 1987; Wild et al. 1990).

So far, the metabolism of AFB_1_ is already relatively well described, but comprehensive quantitative data covering a broad spectrum of metabolites are still lacking. Therefore, the present investigation focused on species differences in AFB_1_ metabolism between rats, mice, and humans using primary hepatocytes. The absolute quantification of main metabolites, in addition to the analysis of DNA and protein adducts at specific time points, gives a comprehensive metabolism kinetics of AFB_1_ in these species. The synthesis of reference compounds enabled the quantification of AFB_1_ adducts, which are not commercially available.

## Materials and methods

### Chemicals and reagents

Solvents and chemicals were purchased from Fisher Scientific (Schwerte, Germany), Carl Roth (Karlsruhe, Germany), Sigma-Aldrich (Steinheim, Germany), or VWR (Darmstadt, Germany) if not further specified. Water was purified via the PureLab Flex2 system (Veolia Water Technologies, Celle, Germany). AFB_1_ (purity ≥ 98%, Sigma-Aldrich), AFM_1_ (purity ≥ 95%, Enzo Life Sciences, Lörrach, Germany), AFP_1_ (TRC, Toronto, Canada), AFQ_1_ and AFL (purity > 98%, Biomol, Hamburg, Germany) were commercially available as reference compounds and their concentration was adjusted gravimetrically. Solvents were used in LC–MS grade.

### Synthesis and purification of reference compounds

#### AFB_1_-epoxide

The epoxidation of AFB_1_ was induced by a reaction with dimethyldioxirane (DMDO), which was synthesized according to a previously reported method based on the reaction of acetone with potassium peroxymonosulfate (Taber et al. 2013; Sass et al. 2013). The concentration of DMDO was determined via iodometric titration just before use. 0.06 mmol of AFB_1_ was dissolved in 3 mL of dichlormethane and 0.09 mmol of DMDO was added. The solution was stirred for 15 min at room temperature and the solvent as well as the remaining DMDO were evaporated afterwards. The residue, containing the AFB_1_-epoxide, was reconstituted in 1.1 mL dimethylsulfoxide (DMSO).

### AFB_1_-*N*^7^-guanine

For the synthesis of AFB_1_-*N*^7^-guanine (AFB_1_-Gua), based on previous reports (Vidyasagar et al. 1997), 0.12 mmol guanine was added to the solution containing 0.06 mmol of AFB_1_-epoxide in 1.1 mL DMSO and stirred for 15 min at room temperature. The product was purified using a Bond Elute PRS solid phase extraction (SPE) cartridge (1 mg, 6 mL, Agilent, Waldbronn, Germany). After washing with 2% formic acid (FA) and pure methanol (MeOH), the compound was eluted with 5% ammonium hydroxide in MeOH. The adduct was purified using preparative high-performance liquid chromatography coupled to an ultraviolet detector (HPLC–UV) (365 nm) on a Nucleodur Phenyl-Hexyl column (250 × 4 mm, 5 µm, Macherey–Nagel, Düren, Germany) and an acetonitrile (ACN)/water gradient supplemented with 0.1% FA. Because of the low yield of the synthesis the concentration of the final solution was determined photometrically based on a molar extinction coefficient of 18.000 L/mol/cm (362 nm) in 0.05 M phosphate buffer (pH 7.0) (Coskun et al. 2019). Further details on the purification steps are shown in Online Resource 1 (Tables S1 and S2).

### AFB_1_-formamidopyrimidine

Parts of the purified AFB_1_-Gua were used to synthesize the AFB_1_-FAPY guanine (Alekseyev et al. 2004). Therefore, an aliquot of the AFB_1_-Gua solution was alkalized to pH 10–11 with 0.1 M sodium hydroxide (NaOH) and shaken for 4 h at 37 °C. Because of the low amounts of starting material, just a qualitative standard was synthesized without further purification.

### AFB_1_-lysine

The synthesis of AFB_1_-Lys was carried out in parallel to the AFB_1_-Gua synthesis. The two washing phases from the SPE clean-up of AFB_1_-Gua were used separately as starting material as the lysine adduct is formed from AFB_1_-dialdehyde and AFB_1_-dihydrodiol which eluted in these steps. The first washing phase was diluted with phosphate buffer and the second washing phase was evaporated to dryness and the residue was also reconstituted in phosphate buffer (0.1 M, pH 7.4). Two equivalents of *N*_α_-Boc-l-lysine (0.12 mmol, Alfa Aesar, Kandel, Germany), protected at the α-amino group, were added to both solutions and stirred at room temperature in the dark for about 22 h.

The samples were purified on a Strata-X (1 g, 12 mL, Phenomenex, Aschaffenburg, Germany) SPE cartridge and AFB_1_-Boc-lysine was eluted with water and 50% MeOH during the washing steps. For further preparative purification using HPLC–UV (399 nm), a Nucleodur Phenyl-Hexyl column (250 mm × 4 mm, 5 µm) and a gradient of ACN and water with 0.1% FA were used. The Boc-protection group was removed by the addition of trifluoracetic acid followed by 18 h of stirring at room temperature in the dark. For the neutralization of the solution phosphate buffer (0.1 M, pH 7.4) and 10 M NaOH were added. For further purification, the same preparative HPLC method as applied for the purification of the Boc-lysine adduct was used. The concentration of the purified solution in 0.1 M phosphate buffer (pH 7.4) was determined photometrically with a molar extinction coefficient of 30 866 L/mol/cm (400 nm) (Renaud et al. 2022). A purity of 59.5% was determined using HPLC–DAD (diode-array detector). More details on the purification can be found in Online Resource 1 (Tables S3 and S4).

### AFB_1_-***N***-acetylcysteine

The *N*-acetylcysteine derivative was formed by a reaction of AFB_1_-epoxide with *N-*acetylcysteine. Therefore, 20 equivalents of *N*-acetylcysteine (0.64 mmol, Alfa Aesar) were dissolved in phosphate buffer (0.1 M, pH 7.4), cooled to 5 °C and added to 0.032 mmol of AFB_1_-epoxide within 10 min. The solution was stirred for 60 min at 5 °C. The included acetone was evaporated and the residue was purified with an SPE clean-up on a C18-E cartridge (2 g, 12 mL, Phenomenex). AFB_1_-*N*-acetylcysteine (AFB_1_-NAC) eluted with 10–20% of MeOH and the organic solvent of the combined phases was removed within 30 min at 30 °C *in vacuo* as the compound degrades easily. The remaining solution was lyophilized and further purified via preparative HPLC–UV (350 nm) on a Nucleodur Phenyl-Hexyl column (250 mm × 10 mm, 5 µm). A second purification step with a similar column but with a lower diameter (250 × 4 mm, 5 µm) was carried out afterward. In the following SPE purification with a Strata -X-33 µm (100 mg, 3 mL, Phenomenex), the compound was eluted with 100% of MeOH, and the solution was directly used for the photometric quantification on the base of a molar extinction coefficient of 20 000 L/mol/cm (Moss et al. 1985). A purity of 100% was determined using HPLC–DAD (365 nm). Further details are shown in Online Resource 1 (Tables S5 and S6).

### AFB_1_-glutathione

The glutathione derivative was synthesized enzymatically using rat liver microsomes, and cytosol as initial experiments indicated higher yields of AFB_1_-glutathione compared to the non-enzymatic reaction of glutathione (GSH) with AFB_1_-epoxide. A solution containing 5 mM GSH, 0.5 mM AFB_1_, and 0.1 M phosphate buffer (pH 7.4) (similar to Moss et al. 1983) was supplemented with rat liver microsomes and rat liver cytosol to protein contents of 5.25 mg/mL and 7.9 mg/mL, respectively. Furthermore, 10 mM glucose 6-phosphate, 664 µM nicotinamide adenine dinucleotide phosphate (NADP^+^), and 2 U/mL glucose 6-phosphate dehydrogenase were used. The reaction was carried out in 62 preparations of 1 mL each, as an upscaling reduced reaction yield. The suspension was shaken for 4 h at 37 °C followed by the addition of two equivalents of ice-cold ACN to stop the reaction. The proteins precipitated and the samples were centrifuged at 3000 × g at 20 °C. An SPE clean-up of the upper phase on a Strata C18-E SPE cartridge (2 g, 12 mL, Phenomenex) followed and the adduct was eluted with 20–30% of MeOH. The compound was further purified using preparative HPLC–UV (360 nm) on a Nucleodur Phenyl-Hexyl column (250 mm × 4 mm, 5 µm). The purified product was quantified photometrically in 0.1 M phosphate buffer (pH 7.4) based on an extinction coefficient of 21 800 L/mol/cm (Degen and Neumann 1978; McHugh et al. 1996). A purity of 100% was determined using HPLC–DAD (360 nm). Further details are presented in Online Resource 1 (Tables S7 and S8).

### Characterization of reference compounds

All synthesized and purified reference compounds were characterized based on exact mass and fragmentation spectra, which are shown in Online Resource 1. An HPLC-qToF-MS coupling was used including an Elute pump (Bruker Daltonics, Bremen, Germany), a column oven (Bruker Daltonics) set to 40 °C, and a PAL HTC-xt autosampler (CTC Analytics, Zwingen, Switzerland) cooled to 8 °C. Furthermore, an Impact II with an Apollo II ESI source (Bruker Daltonics) was selected for mass spectrometric detection. The chromatographic separation was carried out on a Nucleodur C18 Pyramid column (100 × 2 mm, 3 µm, Macherey–Nagel) with an ACN/water gradient supplemented with 0.1% FA. The detailed gradient, further mass spectrometric parameters, and fragmentation data are shown in Online Resource 1 (Tables S9 and S10, Figures S1-S5). The same instrument setup and separation method coupled to a DAD (Bruker Daltonics) were used for the purity check.

### Primary hepatocyte culture

Hepatocytes were isolated from male Wistar rats (180–200 g, Charles River, Sulzfeld, Germany) and from 8- to 12-week-old male C57BL/6N mice by using a previously described two-step perfusion procedure (Godoy et al. 2013, Appendix 1). The liver perfusions of rats and mice to isolate hepatocytes were approved by the local authorities. The freshly isolated primary rodent hepatocytes were used for plating on cell culture dishes, precoated with collagen from rat tail tendon, and then kept in sandwich culture as described before in detail (Godoy et al. 2013; Appendix 2). In brief, rat hepatocytes were seeded at a density of 1 × 10^6^ cells/well, and mouse hepatocytes were seeded at 0.85 × 10^6^ cells/well in 6-well plates on collagen from rat tail tendon (Roche, Basel, Switzerland) in 2 mL Williams E medium (Pan-biotech, Aidenbach, Germany), which was supplemented with l-glutamine (PAN-biotech) as well as penicillin, streptomycin, and gentamycin as antibiotics (PAN-biotech). A mixture of insulin, transferrin, and selenium (Sigma-Aldrich) as well as dexamethasone (Sigma-Aldrich) was also added. After 3 h of plating, the second collagen layer was added and incubated for 30 min. After complete gelation of collagen, cell cultures were treated with 1 µM and 10 µM AFB_1_, respectively. The two concentrations were chosen to avoid cytotoxic effects but enable also the detection of metabolites formed in low yields.

Cryopreserved human hepatocytes from three donors purchased from Lonza (Basel, Switzerland) and Biovit (Brussels, Belgium) were also seeded on collagen-coated dishes at a density of 1 × 10^6^ cells/well. 24 h after initial seeding, the human hepatocytes in sandwich culture were incubated with AFB_1_ at 1 µM in triplicate, while two biological replicates were treated with 10 µM AFB_1_.

Culture medium samples of rat, mouse, and human hepatocytes were withdrawn in duplicate at 10 min, 30 min, 60 min, 2 h, 4 h, 6 h, and 24 h after incubation with AFB_1_ and were stored at − 20 °C until HPLC–MS/MS analysis.

### Determination of macromolecular adducts and intracellular metabolites

Additional wells were prepared for the determination of AFB_1_ bound to macromolecules such as the DNA and proteins after 30 min, 2 h, and 24 h. Furthermore, the intracellular content of the analytes was determined in these samples. The isolation of the DNA was performed according to a modified protocol previously reported for the isolation of fungal DNA (Cenis 1992). The cells were washed twice with 2 mL of phosphate-buffered saline prior to adding 200 µL of lysis buffer (200 mM Tris(hydroxymethyl)aminomethane HCl (pH 8.5, Carl Roth), 0.25 M NaCl (Merck, Darmstadt, Germany), 0.025 M EDTA disodium salt (Carl Roth), and 0.5% sodium dodecyl sulfate (Carl Roth). A cell scraper was used for cell harvest and the lysate was transferred into a DNase-free reaction tube. The cells were resuspended and stored at −20 °C until further sample preparation. After thawing, 20 µL of proteinase K (2 mg/mL, Carl Roth) was added for protein digestion. The first replicate was digested at 58 °C for 90 min. As the high temperature turned out to cause adduct degradation, the digestions of the second and the third replicates were performed at room temperature. After protein digestion, 200 µL of potassium acetate (5 M, pH 7, Carl Roth) was added and the samples were centrifuged for 30 min (14,840 × *g*, 4 °C). 1 mL isopropanol (− 20 °C) was added to the supernatant and the samples were inverted until the DNA precipitated and the samples were centrifuged again (30 min, 14,840 × *g*, 4 °C).

The supernatant was diluted by a factor of 5 for the determination of intracellular AFB_1_ and metabolite concentrations. 1 mL of the remaining supernatant was used to determine the formation of protein adducts by release and purification of AFB_1_-lysine following a slight modification of the protocol published by McCoy et al. (2005). Therefore, isopropanol was evaporated and 500 µL of pronase (6.5 mg/mL in phosphate-buffered saline, Merck) supplemented with 10 µL of ^13^C_6_^15^N_2_-AFB_1_-Lys as stable isotope-labeled internal standard (10 ng/mL) was added. The samples were incubated at 37 °C overnight to ensure complete protein digestion. After centrifugation, the supernatant was purified and concentrated via SPE using a 1 cc Oasis MAX cartridge (Waters, Eschborn, Germany). AFB_1_-Lys was eluted with 2% FA in MeOH, evaporated completely, and reconstituted in ACN/water (1 + 19, v/v) for the analysis. The detailed SPE cleanup method is provided in Online Resource 1 (Table S11).

The DNA pellet was washed with 500 µL of 70% ethanol and centrifuged (30 min, 14,840 × *g*, 4 °C). The supernatant was discarded and the DNA pellet was dried using a vacuum concentrator (10 mbar, 10 min, 1750 rpm, 40 °C). 100 µL of digestion buffer (10 mM Tris(hydroxymethyl)aminomethane, 5 mM MgCl_2_, pH 7), which included 5 µL RNase (10 mg/mL, Sigma-Aldrich), was added to each sample and the RNA was digested overnight at 37 °C and gentle shaking. Afterward, 50 µL of potassium acetate was added and the DNA was precipitated with 500 µL isopropanol. Similar to the first precipitation the DNA was washed with 500 µL of 70% EtOH and dried under vacuum. After adding 100 µL of digestion buffer, the DNA was digested by 10 U DNase, 500 mU phosphodiesterase from *Crotalus adamanteus* venom, and 500 mU alkaline phosphatase from bovine intestinal mucosa (all from Sigma-Aldrich) at 37 °C and gentle shaking overnight. Subsequently, 200 µL of ACN was added (− 20 °C) to precipitate the proteins and the samples were centrifuged (15 min, 14,840 × *g*, 4 °C). The supernatant was evaporated to dryness using a vacuum concentrator (10 mbar, 1750 rpm, 40 °C) and reconstituted in 50 µL water. An aliquot of the samples was diluted by a factor of 100 with water for the determination of 2´-deoxyguanosine.

### Analysis of AFB_1_ metabolites

**Fig. 2:**
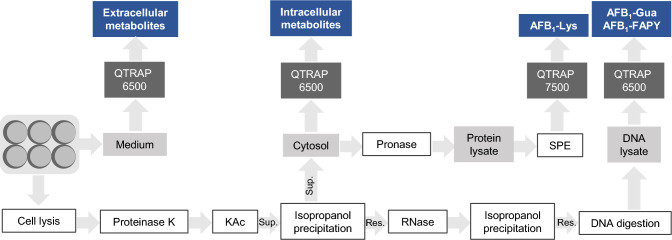
Sample preparation and analyzed metabolites in the various matrices after incubation of AFB_1_ in primary hepatocytes of mouse, rat, and human for 24 h (*Sup* supernatant, *Res* residue, *KAc* potassium acetate, *SPE* solid phase extraction)

All analytes, except intracellular AFB_1_-Lys, were analyzed using HPLC–MS/MS on an Agilent Infinity 1260 HPLC system in combination with a QTRAP 6500 triple quadrupole mass spectrometer (SCIEX, Darmstadt, Germany). The analytes were separated using a Nucleodur C18 Pyramid column (150 mm × 2 mm, 3 µm, Macherey–Nagel). ACN (solvent A) and water (solvent B) both supplemented with 0.1% FA were used for gradient elution. In addition to the metabolites of AFB_1_, 2´-deoxyguanosine (dG) was quantified in the DNA lysates to normalize AFB_1_-Gua formation on DNA yield. AFB_1_-Lys was analyzed using a QTRAP 7500 mass spectrometer (Sciex) in combination with an Agilent Infinity II Bio HPLC system. AFB_1_-Lys was separated on a Reprosil-Pur C18 AQ HPLC column (150 × 2 mm, 3 µm, Dr. Maisch, Ammerbuch, Germany), also using an ACN/water gradient supplemented with 0.01% acetic acid. The injection volume and the dilution of the samples were selected according to the expected concentrations of the analytes. The samples were diluted with ACN/water (1 + 19, v/v). Detailed quantification data as well as further information on HPLC and MS methods are summarized in the Online Resource 1 (Table S12-S20). Not detectable signals were set to zero for quantification. An overview of sample preparation procedures and the determination of metabolites in the different matrices is shown in Fig. 2.

Formation rates shown in Table S21 were calculated as a mean of the quotients of molar quantity and time. Only the time points representing a linear formation were included.

## Results

The metabolism of AFB_1_ was compared in three different mammalian systems: human, mouse, and rat. First, this study focused on the comparative analysis of the metabolism of AFB_1_ in hepatocytes from two different rodent species. Primary hepatocytes from rats and mice were freshly isolated and treated with 1 µM and 10 µM of AFB_1_ for up to 24 h. Additionally, cryopreserved human hepatocytes were seeded and treated similarly. To study the formation and kinetics of AFB_1_ metabolites, the culture medium was sampled over a time course up to 24 h and analyzed by HPLC–MS/MS. Furthermore, the DNA and the intracellular proteins were isolated and digested after 30 min, 2 h, and 24 h for the analysis of DNA (AFB_1_-Gua and AFB_1_-FAPY) and protein (AFB_1_-Lys) adducts.

Phase II metabolites of AFB_1_ were not commercially available and were prepared from AFB_1_ as references for quantification by HPLC–MS/MS. AFB_1_-GSH was produced enzymatically with rat liver microsomes and cytosol. AFB_1_-NAC, AFB_1_-Lys, and AFB_1_-Gua were prepared following a chemical epoxidation of AFB_1_ with DMDO.

### Analysis of AFB_1_ in the culture medium of the hepatocytes

**Fig. 3:**
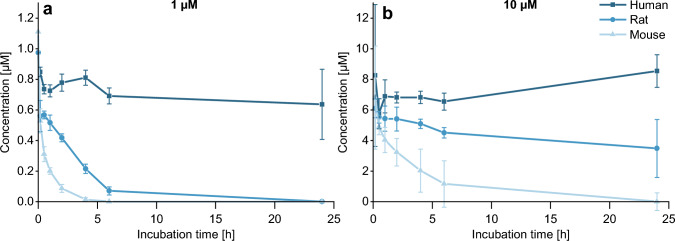
AFB_1_ concentration in mouse (light blue), rat (blue), and human (dark blue) hepatocyte medium after incubation of **a** 1 µM and **b** 10 µM AFB_1_ over 24 h. Quantification was performed in a culture medium using HPLC–MS/MS in *n* = 3 × 2. The 10 µM incubation of human hepatocytes was carried out in *n* = 2 × 2

The detected concentrations of AFB_1_ in the hepatocyte cell culture media over the time course of 24 h are summarized in Fig. 3.

AFB_1_ levels declined rapidly in the culture medium of mouse hepatocytes as after one hour of 1 µM incubation only 0.2 µM of AFB_1_ remained and already after 6 h AFB_1_ was no longer detectable. Fast conversion by mouse hepatocytes occurred also at the high concentration (10 μM), but AFB_1_ was only detectable up to 6 h after incubation.

In rat hepatocyte medium AFB_1_ disappeared completely within 24 h after incubation of 1 µM AFB_1_. However, the decrease was slower in rats compared to mice; after 6 h a concentration of 70 nM AFB_1_ remained in the culture medium of rat hepatocytes compared to the complete disappearance in that of mouse hepatocytes at this time point. In line, incubation of 10 µM AFB_1_ did not result in a complete conversion by rat hepatocytes, with 3.48 µM AFB_1_ remaining after 24 h. The concentration of AFB_1_ in human hepatocyte medium decreased comparatively slowly as 636 nM and 8.54 µM remained after incubation of 1 µM and 10 µM of AFB_1_, respectively, after 24 h.

### Analysis of AFB_1_ metabolites in mouse hepatocyte medium

**Fig. 4:**
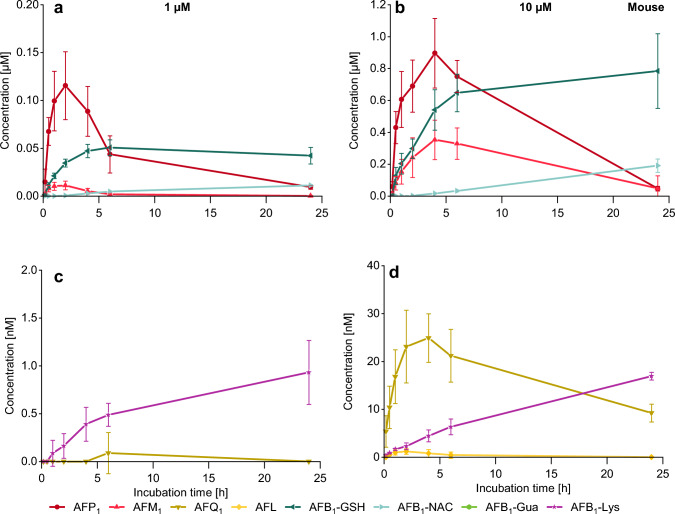
Conversion of AFB_1_ in primary mouse hepatocytes (*n* = 3 × 2) analyzed in cell culture medium over 24 h via HPLC–MS/MS. The diagrams depicted in **a** and **c** relate to an incubation of 1 µM AFB_1_ and **b** and **d** show the kinetics of an incubation of 10 µM AFB_1_. Levels of AFP_1_, AFM_1_, AFB_1_-GSH, and AFB_1_-NAC shown in **a** and **b** are given in micromolar scale; those of the minor metabolites AFQ_1_, AFL, and AFB_1_-Lys in charts **c** and** d** are shown in nanomolar scale

AFP_1_ was detected as the main metabolite of AFB_1_ in mouse hepatocytes and increased within 2 h to 115 nM, followed by a decrease to 9 nM at 24 h (Fig. 4a). The same trend was observed for AFM_1_ but to a lower extent. A concentration of 11 nM AFM_1_ was reached after 2 h of incubation which then declined to a remaining level of 0.3 nM after 24 h. The 10 µM incubation gave a comparable temporal result as the two-phase I metabolites increased within the first 4 h to a maximum of 896 nM AFP_1_ and 353 nM AFM_1_, followed by a decrease until 24 h (Fig. 4b). The higher concentration of 10 µM also enabled the detection of AFQ_1_ (Fig. 4d) which increased within the first 4 h to a maximum of 25 nM, followed by a decrease similar to the other phase I metabolites. AFL was only detected in traces after incubation of 10 µM AFB_1_ and reached the highest concentration of 1 nM within 2 h, followed by a decrease until the end of the experiment (Fig. 4d). Yet another highly abundant metabolite of AFB_1_ was AFB_1_-GSH which increased rapidly in the first 4 h of incubation and reached maximum concentrations of 42 nM and 784 nM after incubation with 1 µM and 10 µM, respectively, at 24 h (Fig. 4a, b). AFB_1_-NAC, most probably a metabolite of AFB_1_-GSH, increased slowly and reached also a maximum after 24 h with quantified concentrations of 11 nM AFB_1_-NAC and 191 nM AFB_1_-NAC for the incubation with 1 µM AFB_1_ and 10 µM AFB_1_, respectively (Fig. 4a, b). AFB_1_-Lys was detectable in the cell culture medium in low concentrations of up to 0.9 nM and 17 nM after 24 h of 1 µM and 10 µM AFB_1_-treatment of primary mouse hepatocytes, respectively (Fig. 4c, d). AFB_1_-Gua was not detected.

### Analysis of AFB_1_ metabolites in rat hepatocyte medium

**Fig. 5:**
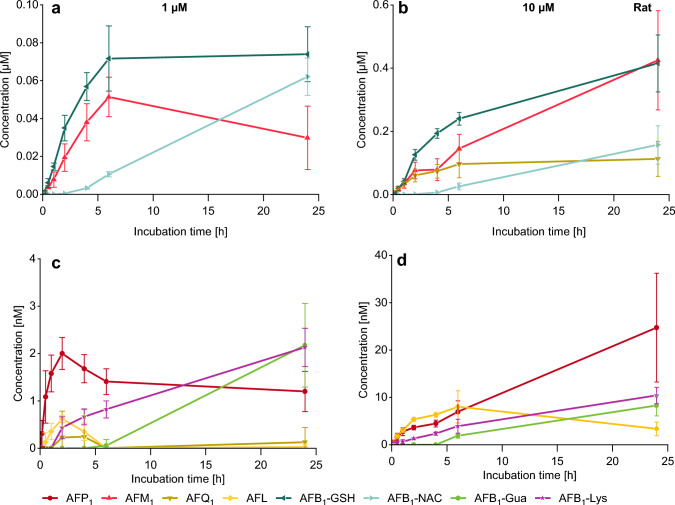
Conversion of AFB_1_ in primary rat hepatocytes (*n* = 3 × 2) analyzed in cell culture medium over 24 h via HPLC–MS/MS. The diagrams depicted in **a** and **c** relate to an incubation of 1 µM AFB_1_ and **b** and **d** show the kinetics of an incubation of 10 µM AFB_1_. Levels of AFM_1_, AFB_1_-GSH, and AFB_1_-NAC are shown in **a** and **b**. AFQ_1_ is also formed in high yields after incubation of 10 µM AFB_1_ as shown in chart **b**, and as a minor metabolite shown in chart **c** after incubation with 1 µM AFB_1_. The other minor metabolites AFP_1_, AFL, and AFB_1_-Lys are shown in charts **c** and **d**

As the main metabolite of rat hepatocytes, AFB_1_-GSH was detected with increasing concentrations, reaching a plateau of 72 nM (1 µM) and 415 nM (10 µM), respectively after 6 h (Fig. 5a, b). These kinetics are similar to mouse hepatocyte metabolism (Fig. 4) while a slower increase was observed for AFB_1_-NAC, especially in the 10 µM incubation. After 24 h incubation, maximum concentrations of only 62 nM and 157 nM of 1 µM and 10 µM, respectively, were reached. The concentration of AFB_1_-NAC was significantly higher in rat hepatocytes in comparison to mouse hepatocytes after 1 µM incubation, whereas the formation after 10 µM incubation was comparable. The main hydroxylated metabolite found in rat hepatocytes was AFM_1_, which was also detected at higher concentrations in rat hepatocyte culture medium compared to the culture medium of mouse hepatocytes. For incubation with 1 µM AFB_1_, a maximum concentration of 51 nM AFM_1_ was determined after 6 h following a slight decline of the AFM_1_ level down to 30 nM after 24 h (Fig. 5a). For incubations with 10 µM AFB_1_, a constant increase of the AFM_1_ concentration in culture medium was observed, amounting to 425 nM after 24 h (Fig. 5b). In the incubations with 10 µM AFB_1_, also a second hydroxylated metabolite AFQ_1_ occurred with a continuously increasing concentration, reaching 112 nM after 24 h (Fig. 5b). The other phase I metabolites AFL and AFP_1_ as well as AFB_1_-Gua and AFB_1_-Lys were detected only in trace amounts (Fig. 5c, d).

### Analysis of AFB_1_ metabolites in human hepatocyte culture medium

**Fig. 6:**
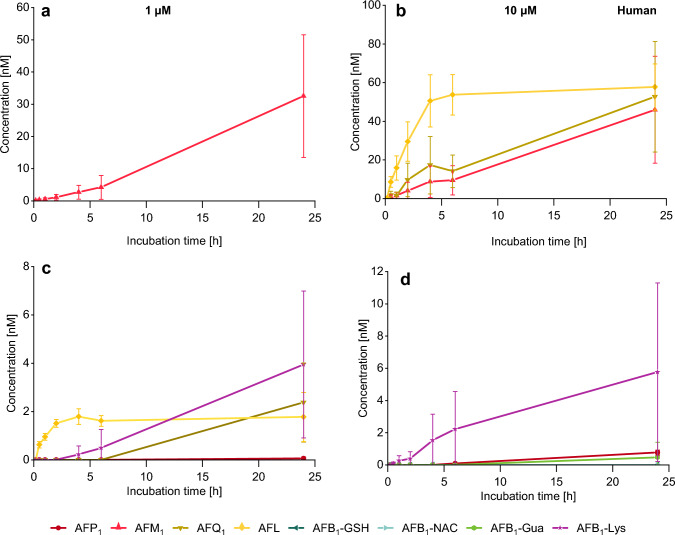
Conversion of AFB_1_ in primary human hepatocytes (*n* = 3 × 2) analyzed in cell culture medium over 24 h via HPLC–MS/MS. The diagrams depicted in **a** and **c** relate to an incubation of 1 µM AFB_1_ and **b** and **d** show the kinetics of an incubation of 10 µM AFB_1_ (*n* = 2 × 2). The major metabolites are given in **a** and **b** and the minor metabolites are depicted in **c** and **d**. Levels of AFM_1_, AFQ_1_, AFL, AFP_1_, AFB_1_-Gua, and AFB_1_-Lys are given in nanomolar scale. AFB_1_-GSH and AFB_1_-NAC were not detected

The main metabolite of AFB_1_ formed by human hepatocytes after 1 µM incubation was AFM_1_ (Fig. 6a). After 24 h, concentrations of this metabolite reached maxima of 33 nM and 46 nM for 1 µM and 10 µM of AFB_1_ which was much lower than the concentrations generated by the other species. In addition, also AFQ_1_ was formed by human hepatocytes. However, after incubation of 1 µM, only 2 nM AFQ_1_ were detectable after 24 h (Fig. 6c). Furthermore, the incubation with 10 µM AFB_1_ strongly induced the formation of AFQ_1_, which was generated continuously up to 52 nM within 24 h (Fig. 6b). AFL is another metabolite which was formed in low concentrations by rodent hepatocytes, increasing up to 50 nM, especially in the first 4 h in human cells (Fig. 6b). Afterward, the concentration remained constant until the end of the incubation period. At the lower concentration, AFB_1_ (1 µM) was metabolized to 2 nM AFL within the first 4 h and remained constant up to 24 h (Fig. 6c). AFP_1_ was just formed in trace amounts. Except for AFB_1_-Lys, no further epoxidation-related adducts were detected. AFB_1_-Lys was found in concentrations of 4 nM (1 µM) and 6 nM (10 µM) in the respective samples, which is comparably high (Fig. 6c, d).

### Formation of adducts with macromolecules

**Fig. 7:**
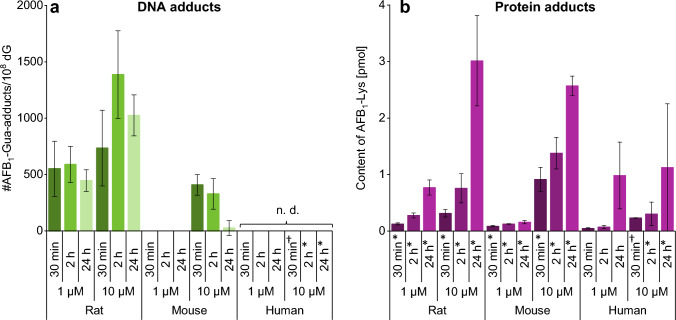
AFB_1_-Gua detected in the DNA **a** and AFB_1_-Lys in proteins **b** after cell lysis at 30 min, 2 h, and 24 h (*n* = 3 × 2). Differing numbers of replicates are marked: *n = 2 × 2, †*n* = 1 × 2. The AFB_1_-Gua data are normalized to the 2’-deoxyguanosine (dG) concentration, *n.d.* not detected

In addition to the determination of metabolites in cell culture media, the amount of DNA adducts still bound to the DNA at specific time points was analyzed in the cell lysates (Fig. 7a). The highest adduct levels were observed in rat hepatocytes. Already after 30 min 550 AFB_1_-Gua/10^8^ dG (1 µM) and 734 AFB_1_-Gua/10^8^ dG (10 µM) were quantified. This level further increased and reached a maximum after 2 h as 1 µM of AFB_1_ resulted in 589 AFB_1_-Gua/10^8^ dG, and the higher concentration led to a number of 1386 AFB_1_-Gua/10^8^ dG. At the end of the experiment, the number of DNA adducts decreased markedly to respective amounts of 446 AFB_1_-Gua/10^8^ dG and 1024 AFB_1_-Gua/10^8^ dG upon incubation of 1 µM and 10 µM AFB_1_ in rat hepatocytes. The formation of the DNA adduct was lower in mouse hepatocytes, and DNA adducts were only detected at the higher test concentration with a maximum after 30 min (408 AFB_1_-Gua/10^8^ dG), followed by a rapid decline by more than 90% between 2 and 24 h (Fig. 7a). In the lysates of human hepatocytes, AFB_1_-Gua was not detected at all.

In addition to the DNA adducts, also the formation of protein adducts was determined in the cell lysates based on the amounts of AFB_1_-Lys after digestion of proteins. The results are summarized in Fig. 7b. Overall, AFB_1_-Lys adduct increased over time, yet contents depended upon species and AFB_1_ concentration. Rat hepatocytes formed 0.8 pmol and 3.0 pmol with 1 µM and 10 µM treatment, respectively, during 24 h incubation. The amounts generated in mouse hepatocytes were different as the 1 µM incubation resulted in less AFB_1_-Lys at all time points reaching a maximum of 0.2 pmol after 24 h. The high substrate concentration resulted in higher amounts of AFB_1_-Lys which were comparable to those in rat hepatocytes or even higher with a maximum of 2.6 pmol after short-term treatment. Human hepatocyte metabolism also led to the formation of AFB_1_-Lys, up to amounts around 1.0 pmol at both AFB_1_ concentrations.

### Determination of intracellular analyte contents

**Fig. 8:**
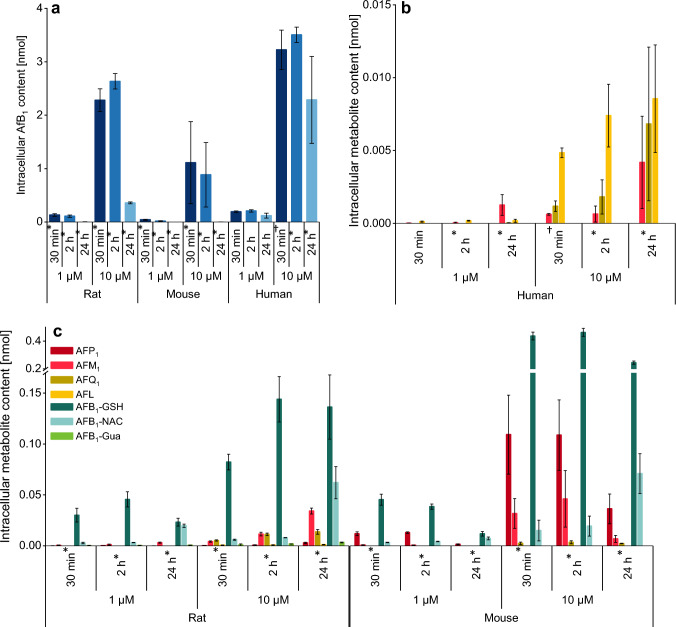
Intracellular contents of AFB_1_ **a** and its metabolites in primary human **b** mouse and rat hepatocytes **c** at 30 min, 2 h, and 24 h in *n* = 3 × 2 numbers of replicates or as marked: *n = 2 × 2 and †*n* = 1 × 2

In addition to the analysis of medium samples also intracellular contents of the analytes were determined (Fig. 8). The data fit the results obtained from the medium samples which showed more rapid metabolism of AFB_1_ at 1 µM and 10 µM by mice than rat hepatocytes and a low conversion by human hepatocytes (Fig. 3). Accordingly, the human hepatocytes contain the highest intracellular amounts of unmetabolized parent compound at all sampling times (Fig. 8a). AFB_1_, the predominant analyte in all samples, was present at high levels over the whole time-course in human hepatocytes, while in rat and mouse the intracellular AFB_1_ content decreased considerably until the end of the experiment. The level of intracellular metabolites increased over time (Fig. 8b, c), and a pattern with some distinct differences between species was observed. For example, AFL, AFQ_1_, and AFM_1_ appeared as major metabolites in human hepatocytes, but AFB_1_-GSH was the major metabolite in rodent hepatocytes, along with AFP_1_ and AFM_1_ in mice, or AFM_1_ and AFQ_1_ in rats. The main metabolites of all species were already present after 30 min regardless of the incubated AFB_1_ concentration. Similar to the medium samples, human hepatocytes only formed low amounts of metabolites, and AFB_1_-GSH was not detected.

## Discussion

The toxicity of AFB_1_ is dependent on bioactivation and detoxication pathways (Fig. 1) and of considerable interest in light of well-known interspecies differences in susceptibility to acute and chronic effects of AFB_1_ (Eaton and Gallagher 1994; Hengstler et al. 1999). The present study has applied advanced analytical methods to elucidate AFB_1_ conversion by hepatocytes of three species to characterize phase I and phase II metabolites in the medium or intracellular as well as macromolecule (DNA, protein) bound products.

### Cellular uptake and conversion rate of AFB_1_

AFB_1_ was transported rapidly into the hepatocytes as the compound occurred intracellularly in high levels in all species already after 30 min (Fig. 8). Based on previously reported volumes of hepatocytes ranging between 3 pL and 6.8 pL (Wiśniewski et al. 2016; Swift et al. 2010; Chatterjee et al. 2014), the intracellular concentrations of AFB_1_ were estimated. In all species, a strong enrichment of up to 1 mM (human cells) compared to the medium concentrations was calculated for the 10 µM AFB_1_ incubation. Hanigan and Laishes (1984) showed a rapid uptake of radiolabeled [^3^H]-AFB_1_ by primary rat and mouse hepatocytes. The intracellular radioactivity increased more rapidly in rat than in mouse hepatocytes and remained constant over 4 h. In contrast, the radioactivity in mouse hepatocytes decreased after 30 min and the maximum level was not as high as in rat hepatocytes (Hanigan and Laishes 1984). The data shown here led to similar conclusions as the maximum cellular AFB_1_ level in mouse hepatocytes was significantly lower than in rat and human cells (Fig. 8a). Furthermore, the intracellular levels of AFB_1_ increased for up to 2 h in rats and in human hepatocytes, while in mouse hepatocytes the highest level was observed at the first determined time-point of 30 min. The decrease in AFB_1_ levels in rodent hepatocytes after 2 h was in line with the formation of intracellular metabolites (Fig. 8c) and an efficient efflux of the metabolites to the cell culture medium. AFB_1_ contents in human hepatocytes decreased slightly between 2 and 24 h while low levels of intracellular metabolites were formed over time (Fig. 8b). A low rate of conversion was also reflected in the culture medium with high remaining AFB_1_ levels up to 24 h (Fig. 3). Notably, (unavoidable) differences in isolation and cultivation procedures may affect hepatocyte viability or metabolic competence. Rodent hepatocytes are prepared from freshly isolated livers by common perfusion procedures (Godoy et al. 2013, Appendix 1). Human liver on the other hand is rarely available and usually removed *post mortem* before hepatocyte isolation, which is most commonly performed from slices of the liver rather than the whole organ. Due to infrequent accessibility, it is more common to cryopreserve human hepatocytes and thaw these cells before the actual experiment. These methodical differences need to be taken into consideration when comparing the quantitative data of biological samples from different species origins. The activity of selected enzymes in primary human hepatocytes is just slightly affected by cryopreservation, but the cultivation time can affect enzyme activity. The activities of CYP 1A2 and 3A4, which catalyze AFB_1_ metabolism in human hepatocytes, are known to decrease with longer adherent cultivation time (Smith et al. 2012; Li et al. 1999; Hewitt et al. 2007; Deng et al. 2018; Wilson et al. 1997; Langouet et al. 1995). Nonetheless, human hepatocytes used in our study were found to form AFL along with AFQ_1_, AFM_1_, AFB_1_-Gua, and AFB_1_-Lys.

Comparing the conversion of AFB_1_ in the three species, the data show that independent of the concentration used (1 µM or 10 µM), the metabolism of AFB_1_ in mice hepatocytes is the fastest followed by rats and humans (Fig. 3). Analysis of AFB_1_ metabolism showed distinct differences in metabolite patterns that confirm and expand knowledge on bioactivation and detoxication pathways in these three species.

### Metabolite pattern of phase I metabolism

In mouse hepatocytes, AFB_1_ was predominantly converted to **AFP**_**1**_ (Fig. 4) in the first hours while the formation of AFP_1_, the *O*-demethylation product of AFB_1_, was significantly lower in the other two species (Figs. 5, 6). Also for other compounds, harmine, scoparone, and 6-methoxy-3-(3′,4′,5′-trimethoxy-benzoyl)-1*H*-indole, a faster *O*-demethylation has been reported for mice compared to rats and humans (Fayyaz et al. 2018; Burke and Upshall 1976; Yao et al. 2007). Fayyaz et al. (2018) suggested CYP2A isoforms to be involved in this demethylation reaction. Early experiments on AFB_1_ metabolism by liver microsomes indicated that the formation of AFP_1_ is preferred in mice compared to rats and humans (Ramsdell and Eaton 1990; Degen and Neumann 1981; Dahms and Gurtoo 1976). The conversion to AFP_1_ is considered as an effective way of detoxifying AFB_1_, as this metabolite is far less toxic shown in fertile chicken eggs and in newborn mice (Buchi et al. 1973; Stoloff et al. 1972). Yet, there were some hints on the formation of AFP_1_-DNA adducts in vivo in rat liver (Croy and Wogan 1981; Essigmann et al. 1982). More recent studies on the toxicity of AFP_1_ are not available and its cytotoxicity on human cells has not been investigated. The highest levels of AFP_1_ were seen after short-term incubation of rodent hepatocytes, followed by a strong decrease (Fig. 4) occurring mainly in mice which is possibly related to further metabolism of AFP_1_ in phase II reactions. In an attempt to identify these conjugated metabolites AFP_1_ glucuronide (AFP_1_-Glc) was detected via HPLC-HRMS in about 100-fold higher levels in the mouse samples than in the rat samples. In human samples no AFP_1_-Glc was detectable. The data are shown in Figure S6 in Online Resource 1. A glucuronidation of AFP_1_ and sulfation have been shown previously (Dalezios et al. 1971; Eaton et al. 1988).

The main phase I metabolite of rat hepatocytes was **AFM**_**1**_ (Fig. 5) which confirms an early report on the presence of AFM_1_ together with AFQ_1_ as major hydroxylated metabolites formed by rat liver post-mitochondrial supernatants (Degen and Neumann 1981). AFM_1_ was also found in cultured mouse and human hepatocytes (Figs. 4, 6) and was also the main metabolite of human hepatocytes treated with 1 µM of AFB_1_. This phase I metabolite occurs also in human and animal milk as well as in dairy products and is a relevant contaminant in human nutrition (Min et al. 2020; Iqbal et al. 2015). AFM_1_ exerts a slightly lower cytotoxic potency than AFB_1_ in various cell systems (Gao et al. 2022; Wu et al. 2021; Green et al. 1982). Therefore, AFM_1_ formation is less effective as a detoxification pathway than conversion to AFP_1_. An epoxidation of AFM_1_ can result in DNA adduct formation and mutagenicity. However, the formation of DNA adducts and genotoxicity of AFM_1_ was lower compared to AFB_1_ (Bujons et al. 1995; Loveland et al. 1988; Lutz et al. 1980; Egner et al. 2003).

Along with AFM_1_ and AFL, **AFQ**_**1**_ was one of the main metabolites of human hepatocytes (Figs. 6, 8b) and was formed mainly at 10 µM incubation of AFB_1_. In contrast to AFM_1_, which is formed by CYP1A2 following Michaelis–Menten kinetics, AFQ_1_ is formed by CYP3A4 which follows Hill kinetics resulting in differences in AFB_1_ metabolite pattern at higher substrate levels (Gallagher et al. 1996; Ueng et al. 1995). According to these models, CYP3A4 is activated only at higher concentrations leading to effective formation of AFQ_1_ at 10 µM AFB_1_ and low activity upon incubation at 1 µM AFB_1_. This effect was observed in rat as well as in human hepatocytes (Figs. 5, 6, 8) and also described previously in human and rat microsomes (Ramsdell and Eaton 1990). In mouse hepatocytes, small amounts of AFQ_1_ were formed upon incubation of 10 µM AFB_1_ (Fig. 4). AFQ_1_ exerts far lower acute toxicity and mutagenicity than AFB_1_, and its carcinogenic potency in rainbow trout is about 1% of AFB_1_ (Eaton and Gallagher 1994). Also with metabolic activation of AFQ_1,_ a mutagenic potency of only 1% relative to AFB_1_ was observed (Wong and Hsieh 1976). Therefore, AFQ_1_ formation was identified as an important detoxification pathway metabolite for humans, as it was found in human hepatocyte incubations (at similar levels as AFM_1_) at high AFB_1_ concentrations. AFQ_1_ has been found in urine and fecal samples along with AFM_1_ and AFB_1_-Gua of Chinese males (Mykkänen et al. 2005). Thus, AFQ_1_ could be used as a biomarker for human AFB_1_ exposure together with AFM_1_, AFB_1_-Gua, and AFB_1_-Lys (Vidal et al. 2018).

Interestingly, **AFL** was formed most efficiently by human hepatocytes and was also the predominant intracellular metabolite at the high (10 µM) incubation concentration (Figs. 6, 8b). AFL is generated by a cytosolic reductase and an interconversion to AFB_1_ is described (Wong et al. 1979). Therefore, this metabolite acts as a reservoir for AFB_1_, which implicates high relevance in toxicity (Rushing and Selim 2019; Wong et al. 1979) as it also binds to DNA in rainbow trout with similar efficiency compared to AFB_1_ (Loveland et al. 1988). Taking into account that AFL is transferred across the placenta and is also formed by placental tissue, this metabolic pathway is of special interest for the developmental toxicity of AFB_1_ (Partanen et al. 2010).

### Bioactivation of AFB_1_ and subsequent reactions

In addition to the above-mentioned phase I reactions, AFB_1_ is epoxidized at the double bond of the furofuran ring. The *exo*-epoxide is known to be responsible for the mutagenicity and cytotoxicity of AFB_1_ due to its high reactivity and binding to DNA and to proteins, with the latter occurring via a reactive AFB_1_-dialdehyde formed from AFB_1_-epoxide (van Vleet et al. 2002; Kärenlampi 1987; Sabbioni et al. 1987). On the other hand, AFB_1_-epoxide can undergo inactivation reactions by phase II enzymes.

The epoxide is detoxified via enzymatic conjugation with glutathione to **AFB**_**1**_**-GSH** (Fig. 1). This metabolite was formed in mouse and rat hepatocytes with comparable kinetics (Figs. 4, 5) despite differences in glutathione S-transferase (GST) isozymes responsible for this reaction (Deng et al. 2018). In contrast, human hepatocytes did not form the AFB_1_-GSH adduct (Fig. 6), which is in line with previous experiments where AFB_1_-epoxide was conjugated with GSH in the presence of mouse cytosol but not with human cytosol (Neal et al. 1998), and in vitro studies with subcellular fractions of rat, mouse, and human origin (Ramsdell and Eaton 1990; Raney et al. 1992a; Moss and Neal 1985). The importance of detoxification via glutathione conjugation was shown by Ilic and colleagues who studied the toxicity of AFB_1_ in mGSTA3 knock-out mice. These mice showed strong hepatic necrosis and had 100-fold higher levels of DNA adducts (AFB_1_-*N*^7^-Gua) than wild-type mice (Ilic et al. 2010). This result is also relevant for humans who have a poor ability to detoxify AFB_1_-epoxide in the liver by glutathione conjugation (Guengerich et al. 1996; Raney et al. 1992b).

The glutathione adduct is apparently further metabolized to **AFB**_**1**_**-NAC** in mouse and rat hepatocytes with a short time delay in relation to AFB_1_-GSH adduct formation. This metabolite has been detected also in isolated rat liver hepatocyte incubations with radiolabeled [^3^H-]AFB_1_ (Ch'ih et al. 1991). Furthermore, some degradation products of AFB_1_-GSH such as AFB_1_-Cys-Gly, AFB_1_-Cys-Gln, and AFB_1_-Cys were identified by HPLC-HRMS in the medium of mice and rat hepatocytes after 24 h culture. The AFB_1_-Cys-Gly metabolite was previously detected in the urine of rats along with other more prominent biomarkers of AFB_1_-exposure (Walton et al. 2001).

To investigate the bioactivation of AFB_1_, DNA was isolated from the cells and the formation of **AFB**_**1**_**-Gua** was determined. The rat hepatocytes formed higher amounts of AFB_1_-Gua compared to mouse and human hepatocytes (Fig. 7a). The adduct level in rat hepatocytes showed an increase between 30 min and 2 h and remained at a high level up to 24 h (at about 70% compared to 2 h). In mouse hepatocytes, the highest level was quantified at 30 min but declined within 24 h to less than 10% of the initial amounts, which indicated efficient removal of AFB_1_-Gua from DNA. Recently, spatio-temporal intravital imaging of AFB_1_ in mice revealed fast uptake from the sinusoidal blood by hepatocytes, and only transient enrichment in hepatocyte nuclei followed by rapid clearance into bile canaliculi (Ghallab et al. 2021). This observation could be related to differences in metabolism and faster DNA repair activity of mouse hepatocytes compared to rats (Bedard et al. 2005). In human hepatocytes, no DNA adducts were detectable in the DNA lysates; however, bioactivation was nevertheless indicated by small amounts of AFB_1_-Gua in the medium (Fig. 6d). Considering the rather low conversion rate by human hepatocytes in this study, the presence of this product in the culture medium and also AFB_1_-Lys adduct formation (Figs. 6, 7) are clear evidence for AFB_1_-epoxidation. Cole et al. (1986) compared DNA adduct formation in primary hepatocytes from mice, rats, and human origin incubated with 200 nM [^3^H-]AFB_1_ and compared its binding to DNA with liquid scintillation counting. In contrast to the data shown here, they could not differentiate between formed adducts. Furthermore, cultivation procedures differed between species, and differences in conversion rates of AFB_1_ need also to be taken into consideration. Nevertheless, they also measured the highest DNA binding in hepatocytes of male rats and only small amounts in mice. The difference between the two rodent species is in line with the data presented here. The DNA adduct formation in rats and mice was reported in numerous publications and the carcinogenicity of AFB_1_ was already described to be far higher for rats than for mice (Woo et al. 2011; Coskun et al. 2019; Croy et al. 1978).

The AFB_1_-epoxide can be hydrolyzed to AFB_1_-dihydrodiol, formed spontaneously or catalyzed by epoxide hydrolases, and epoxide hydrolysis led to some reduction in the genotoxicity of AFB_1_ (Johnson et al. 1997). The AFB_1_-dihydrodiol is unstable and can rearrange pH-dependently to AFB_1_-dialdehyde which reacts with the ɛ-amino group of lysine residues in proteins (see Fig. 1) (Sabbioni et al. 1987). This product was determined in the present investigation as **AFB**_**1**_**-Lys** after protein digestion (Fig. 7b). Its presence in all three species already after short incubation shows that all hepatocytes are capable of an efficient epoxidation of AFB_1_. Species and time-dependent differences in AFB_1_-Lys content may be related to differences in the expression and activity of epoxide hydrolases in mice, rats, and humans (Thomas et al. 1990). Moreover, GSH-conjugation of AFB_1_ epoxide is likely to affect the fraction which is hydrolyzed and then results in the formation of protein adducts. While all metabolites of AFB_1_ were generated at rather low levels in human hepatocytes, AFB_1_-Lys formation was of similar magnitude as in rodent hepatocytes. This result can be related to missing GSH conjugation in human cells and an effective enolization of AFB_1_-epoxide. Because of an accumulation of bioactivated AFB_1_ in proteins, AFB_1_-Lys is also used as a long-term biomarker of human aflatoxin exposure (Sabbioni et al. 1987; Wild et al. 1990; Turner et al. 1998).

Studies on the cytotoxicity of AFB_1_ in rodent species showed that rat hepatocytes are more sensitive than mouse hepatocytes and accumulate also far more [^3^H-]AFB_1_ bound to macromolecules (Hanigan and Laishes 1984). This finding can be now interpreted better considering the new data presented here on the rapid metabolism of AFB_1_ in mouse hepatocytes, mainly to AFP_1_ which is less toxic, an efficient DNA repair of AFB_1_-Gua, and efficient detoxification of the epoxide via glutathione conjugation. Rat hepatocytes metabolize AFB_1_ slower, mainly to AFM_1_ and AFQ_1_. While AFB_1_-epoxide is also converted to AFB_1_-GSH, the levels of DNA adduct and protein binding are higher in rat than in mouse. A considerable part of incubated AFB_1_ was not recovered, which might be due to the further metabolism of phase I metabolites such as glucuronidation of AFP_1_ and degradation of AFB_1_-GSH. Other reported metabolites of AFB_1_ such as AFB_2a_, aflatoxicol M_1_, or aflatoxicol H_1_ (Unger et al. 1977; Salhab and Hsieh 1975; Salhab et al. 1977) as well as AFB_1_-dihydrodiol, dialdehyde, and dialcohol were not quantified in this study.

**Fig. 9:**
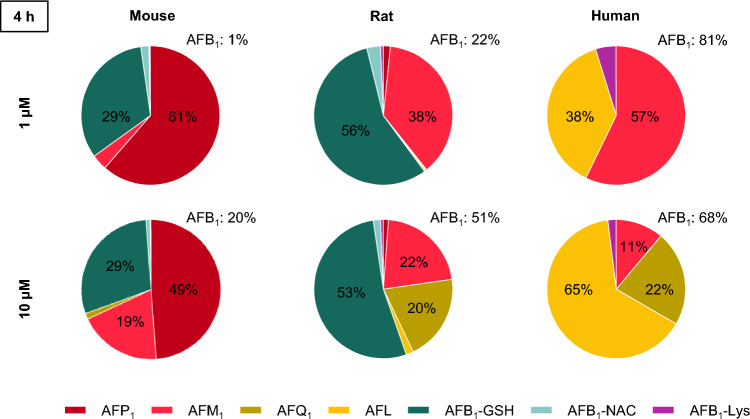
Metabolite pattern and remaining AFB_1_ calculated according to the results in cell culture medium after the incubation of mouse, rat, and human hepatocytes with 1 µM and 10 µM AFB_1_ for 4 h

The distinct metabolite patterns, including human hepatocytes, are summarized in Fig. 9 and reflect different enzyme activities involved in phase I and phase II metabolism of AFB_1_ not only in different species but also in different concentrations. For easier comparison of the kinetics, the formation rates of all compounds in the cell culture medium were calculated and are presented in Table S21 in Online Resource 1.

## Conclusions

The species-specific differences in susceptibility towards AFB_1_ treatment were related to variations in metabolism in previous studies. Therefore, a comprehensive study on phase I and phase II metabolism of AFB_1_ as well as the binding to macromolecules in mouse, rat, and human primary hepatocytes was conducted using HPLC–MS/MS analysis.

The fastest conversion of AFB_1_ was observed in mouse hepatocytes which formed AFP_1_ as the main hydroxylated metabolite with some qualitative evidence for its glucuronidation. Efficient glutathione conjugation of the epoxide contributed to comparatively low binding to DNA and proteins, which is in line with better repair of DNA lesions and low toxicity of AFB_1_ in this species. In contrast, rat hepatocytes formed high amounts of protein and DNA adducts although glutathione conjugation appeared to be similar to mice. Also, AFM_1_ which exerts higher toxicity than AFP_1_ was detected as a major phase I metabolite in rat hepatocytes as well as in human cells. The formation of AFQ_1_ was observed at high substrate concentrations in rat and human hepatocytes. AFL occurs mainly in human cells and is probably of high relevance for toxicity. Missing or poor detoxication by glutathione conjugation favored DNA and protein adduct formation in accord with the known high toxicity and mutagenicity in the human liver. The analysis showed that the overall metabolite pattern of the three species is hardly comparable. Moreover, it was demonstrated that in addition to glutathione conjugation, also phase I metabolism significantly contributes to detoxification and species susceptibility of AFB_1_.

The comprehensive analysis using advanced methodology confirms early results on the metabolism of AFB_1_. Because of the high sensitivity and selectivity of HPLC–MS/MS measurements, more specific insights were obtained into the toxification and detoxification pathways of AFB_1_. This is the first study comparing the metabolism of AFB_1_ comprehensively in mice, rats, and humans in primary hepatocytes considering the quantification of a multitude of metabolites as well as the binding of AFB_1_ to macromolecules.

### Supplementary Information

Below is the link to the electronic supplementary material.

Supplementary file1 (PDF 345 KB)

## Data Availability

Online Resource 1 includes all relevant additional data which are not shown in the publication.
