# Supplementary material for: Comparative metabolism of aflatoxin B1 in mouse, rat and human primary hepatocytes using HPLC–MS/MS
Source: Arch Toxicol. 2023 Oct 5;97(12):3179–96. doi: 10.1007/s00204-023-03607-z (PMC10567917; doi:10.1007/s00204-023-03607-z)
Supplement: Supplementary file 1 — Supplementary file1 (PDF 345 KB) [file 204_2023_3607_MOESM1_ESM.pdf]

Supplementary material
for

Comparative metabolism of aflatoxin B_1 in mouse, rat and human primary hepatocytes using HPLC-MS/MS

Andrea Gerdemann[1] (andrea.gerdemann@uni-muenster.de), Benedikt Cramer[1], Gisela H. Degen[2], Jannik Veerkamp[1], Georgia Günther[2], Wiebke Albrecht[2], Matthias Behrens[1], Melanie Esselen[1], Ahmed Ghallab[2, 3], Jan G. Hengstler[2], Hans-Ulrich Humpf[1*] (humpf@uni-muenster.de)

[1]Institute of Food Chemistry, University of Münster, Corrensstraße 45, 48149 Münster, Germany

[2]Leibniz Research Centre for Working Environment and Human Factors (IfADo), Ardeystraße 67, 44139 Dortmund, Germany

[3]Department of Forensic Medicine and Toxicology, Faculty of Veterinary Medicine, South Valley University, 83523 Qena, Egypt.

Corresponding Author:

*Prof. Dr. Hans-Ulrich Humpf, Institute of Food Chemistry, 48149 Münster, Germany; e-mail address: humpf@uni-muenster.de

Purification of the reference compounds

AFB$_1$-N^7-guanine

Table S1: SPE purification parameters for AFB$_1$-Gua.

SPE cartridge	Bond Elut PRS (1 mg, 6 mL)
Conditioning	6 mL 0.1 % FA in MeOH
Equilibration	6 mL 2 % FA
Washing 1	9 mL 2 % FA
Washing 2	12 mL MeOH
Elution	2 × 6 mL 5 % NH_4OH in MeOH

Table S2: Preparative HPLC purification parameters for AFB$_1$-Gua.

**Column**	Nucleodur Phenyl-Hexyl (250 × 4 mm, 5 µm), Macherey-Nagel		
**Detection**	UV (365 nm)		
**Time [min]**	**Flow Rate [mL/min]**	**ACN + 0.1 % FA [%]**	**H_2O + 0.1 % FA [%]**
1	1.5	95	5
7	1.5	85	15
20	1.5	85	15
30	1.5	5	95
35	1.5	5	95
35.1	1.5	95	5
45	1.5	95	5

AFB_1-Boc-lysine and AFB_1-lysine

Table S3:SPE purification parameters for AFB_1-Boc-lysine.

**SPE cartridge**	**Strata X (1 g, 12 mL), Agilent**
**Conditioning**	6 mL MeOH
**Equilibration**	6 mL H_2O
**Washing 1**	6 mL H_2O
**Washing 2**	6 mL MeOH/H_2O (1+1, *v*/*v*)
**Washing 3**	12 mL MeOH
**Elution**	2 × 6 mL 2 % FA in MeOH

Table S4: Preparative HPLC purification parameters for AFB_1-Boc-lysine and AFB_1-lysine.

**Column**	Nucleodur Phenyl-Hexyl (250 × 4 mm, 5 µm), Macherey-Nagel		
**Detection**	UV (399 nm)		
**Time [min]**	**Flow Rate [mL/min]**	**ACN + 0.1 % FA [%]**	**H_2O + 0.1 % FA [%]**
0	1.5	5	95
55	1.5	95	5
60	1.5	95	5
60.1	1.5	5	95
70	1.5	5	95

AFB$_1$-*N*-acetylcysteine

Table S5: SPE purification parameters for AFB$_1$-*N*-acetylcysteine.

**SPE cartridges**	**Strata C18-E (2 g, 12 mL), Agilent**	**Strata-X 33 µm (100 mg, 3 mL), Agilent**
**Conditioning**	10 mL MeOH	1 mL MeOH
**Equilibration**	10 mL H_2O	2 mL H_2O
**Washing**	10 mL H_2O	-
**Elution**	10 mL 10 % MeOH	1 mL MeOH
	10 mL 20 % MeOH	
	10 mL 30 % MeOH	
	10 mL 50 % MeOH	
	10 mL 70 % MeOH	
	10 mL 100 % MeOH	

Table S6: Preparative HPLC purification parameters for AFB$_1$-*N*-acetylcysteine.

**1. Purification**				**2. Purification**			
**Column**	Nucleodur Phenyl Hexyl (250 × 10 mm, 5 µm), Macherey-Nagel			**Column**	Nucleodur Phenyl Hexyl (250 × 4 mm, 5 µm), Macherey-Nagel		
**Detection**	UV (350 nm)			**Detection**	UV (350 nm)		
**Time [min]**	**Flow Rate [mL/min]**	**ACN + 0.1 % FA [%]**	**H_2O + 0.1 % FA [%]**	**Time [min]**	**Flow Rate [mL/min]**	**ACN + 0.1 % FA [%]**	**H_2O + 0.1 % FA [%]**
0	5	5	95	0	1.5	5	95
10	5	20	80	5	1.5	5	95
30	5	45	55	5	1.5	13	87
31	5	95	5	30	1.5	35	65
40	5	95	5	31	1.5	95	5
41	5	5	95	40	1.5	95	5
50	5	5	95	41	1.5	5	95
				50	1.5	5	95

AFB$_1$-GSH

Table S7: SPE purification parameters for AFB$_1$-GSH.

SPE cartridge	Strata C18-E (2 g, 12 mL), Agilent
Conditioning	10 mL MeOH
Equilibration	10 mL H_2O
Washing	10 mL H_2O
Elution	10 mL 10 % MeOH
	10 mL 20 % MeOH
	10 mL 30 % MeOH
	10 mL 50 % MeOH
	10 mL 70 % MeOH
	10 mL 100 % MeOH

Table S8: Preparative HPLC purification parameters for AFB$_1$-GSH.

Column	Nucleodur Phenyl-Hexyl (250 × 4 mm, 5 µm) Macherey-Nagel		
Detection	UV (399 nm)		
Time [min]	Flow Rate [mL/min]	ACN + 0.1 % FA [%]	H_2O + 0.1 % FA [%]
0	1.5	5	95
55	1.5	95	5
60	1.5	95	5
60.1	1.5	5	95
70	1.5	5	95

Characterization of reference compounds using HPLC-qToF-MS

Table S9: Analysis parameters for the characterization of reference compounds.

**Column**	Nucleodur Pyramid (100 × 2 mm, 3 µm), Macherey-Nagel		
**Detection**	HPLC-HRMS (Table S10)		
**Time [min]**	**Flow Rate [mL/min]**	**ACN + 0.1 % FA [%]**	**H_2O + 0.1 % FA [%]**
0	0.5	5	95
2	0.5	5	95
10	0.5	95	5
12	0.5	95	5
12.2	0.5	5	95
15	0.5	5	95

Table S10: Mass spectrometric parameters for the characterization of reference compounds.

**Mass spectrometer**	qTOF (Impact II)	
**ESI source**	Apollo II	
**Source parameters**	End Plate Offset	500 V
	Capillary	4500 V
	Nebulizer	2 bar
	Dry gas	10 L/min
	Dry temperature	220 °C
	Ion polarity	Positive
**Fragmentation parameters**	Spectra range	*m/z* 30-1000
	Spectra rate	6 Hz
	**Auto MS/MS mode**	
	Number of precursors	2
	Absolute threshold (1000 sum.)	200 cts.
	Exclude after	3 Spectra
	Release after	0.5 min
	Smart exclusion	3 ×

HPLC-qTOF-MS fragmentation spectra of reference compounds

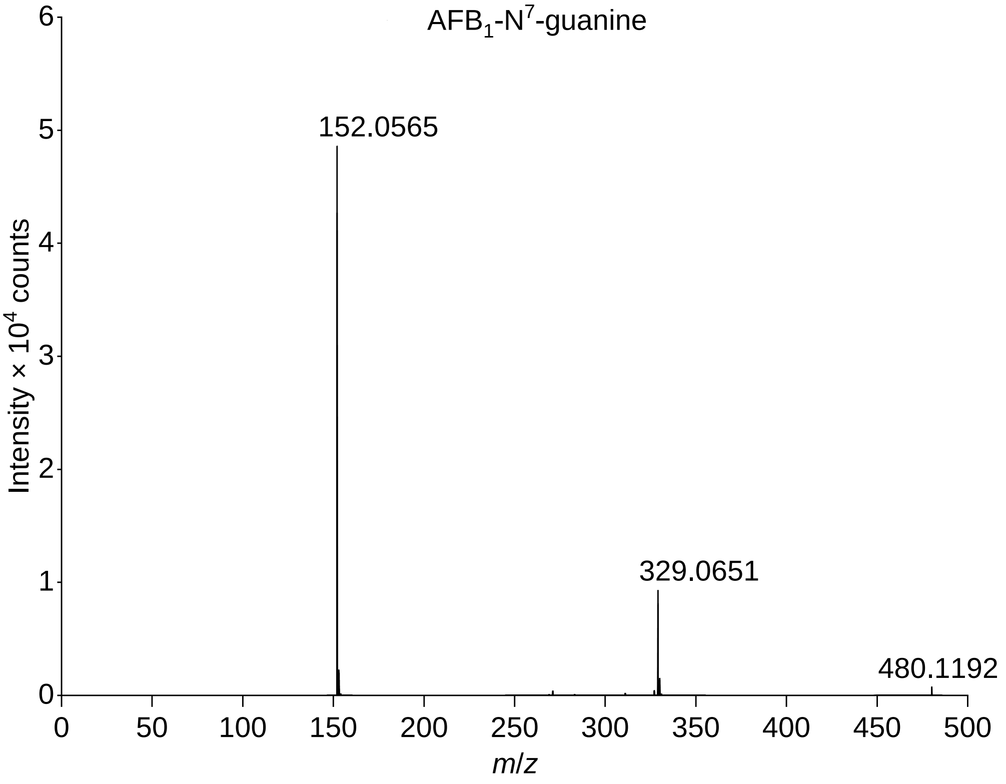

Figure S1: Fragmentation spectrum of synthesized AFB_1-N^7-guanine reference.

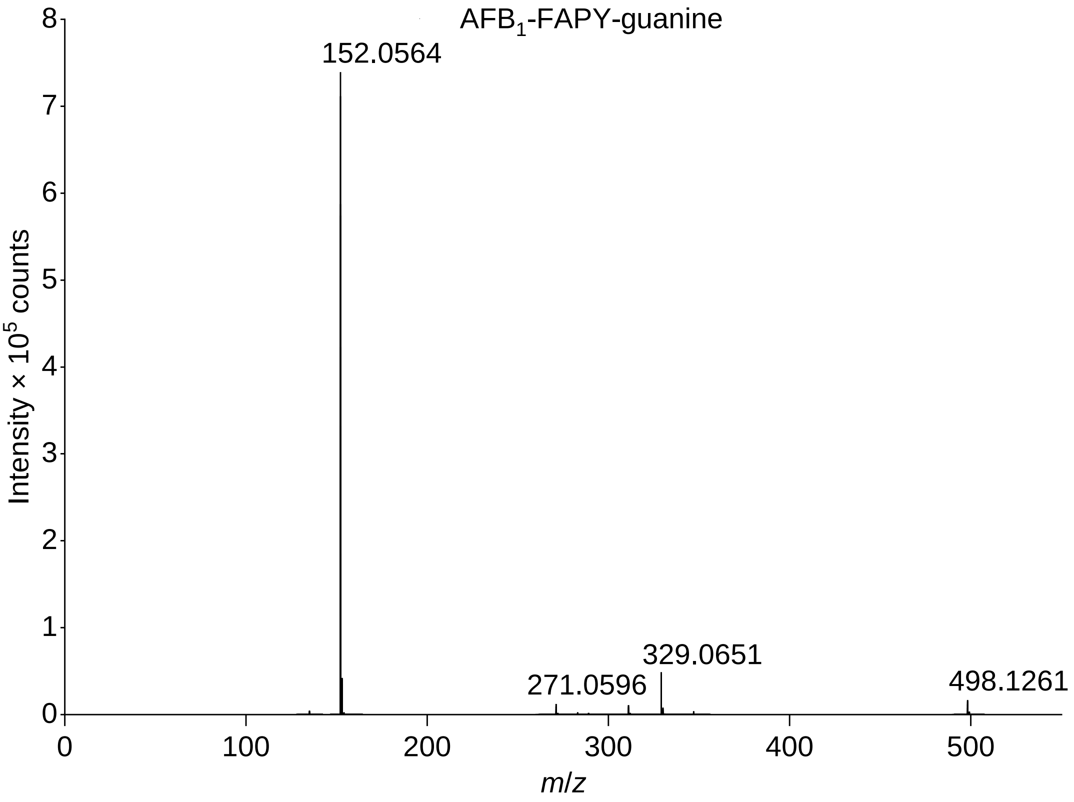

Figure S2: Fragmentation spectrum of synthesized AFB_1-FAPY-guanine reference.

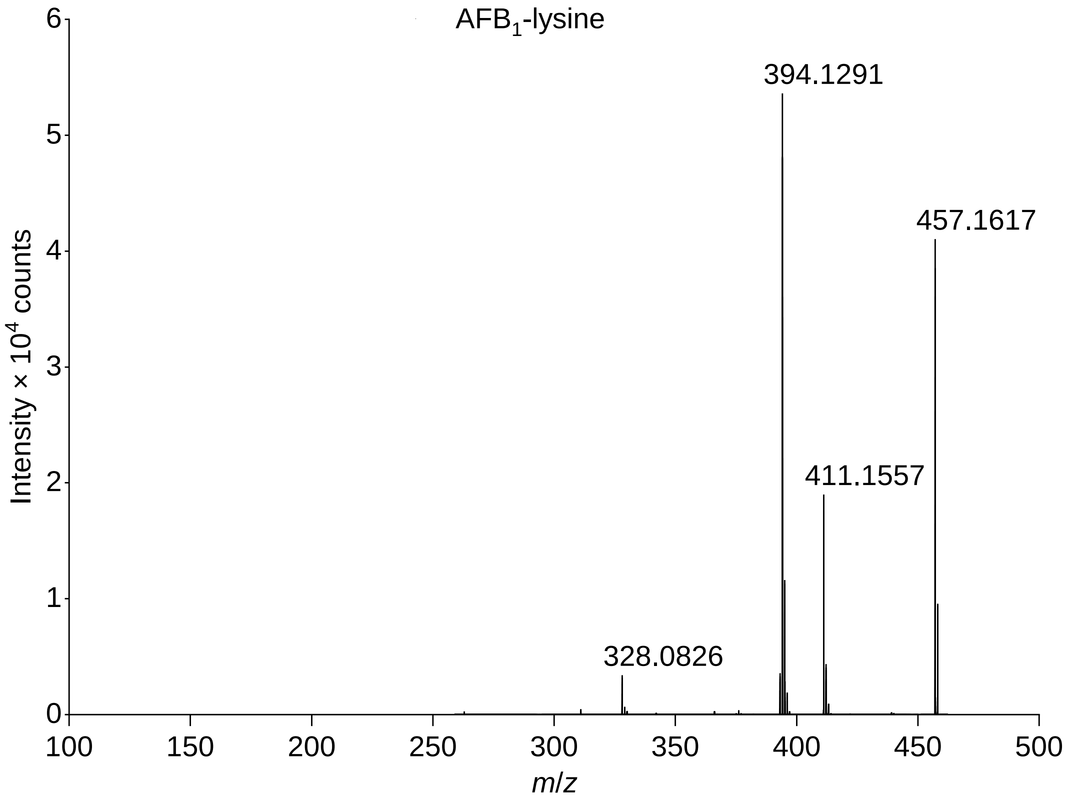

Figure S3: Fragmentation spectrum of synthesized AFB_1-lysine reference.

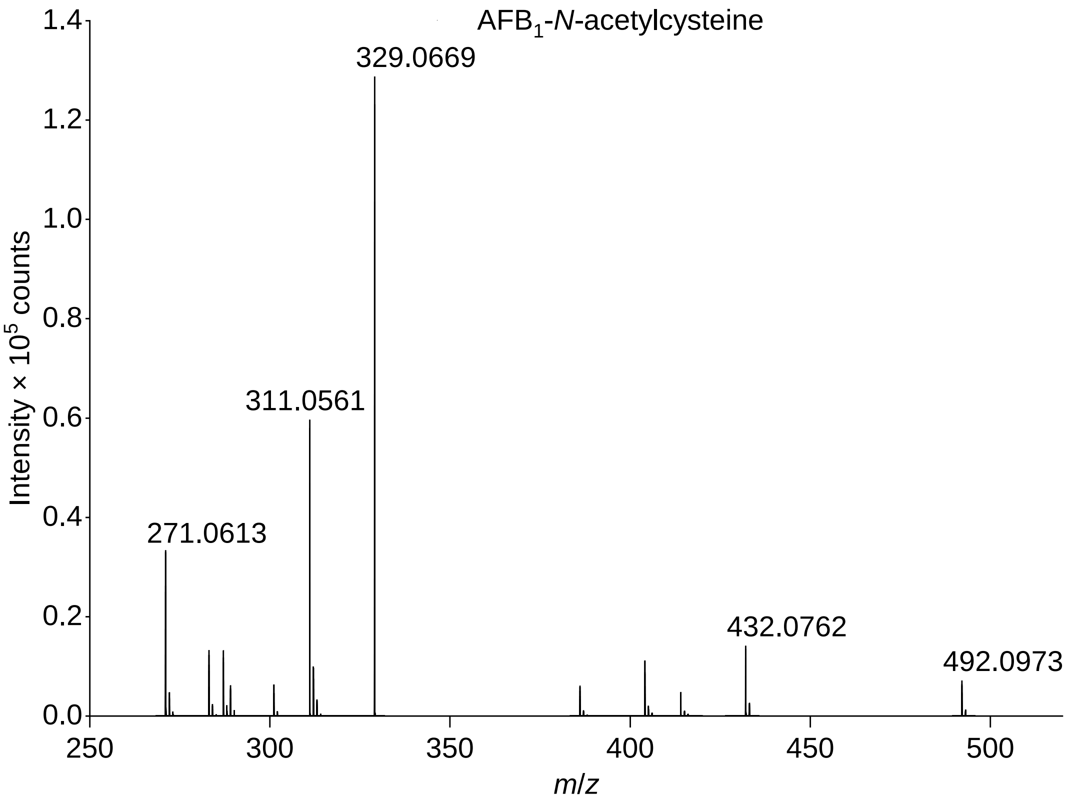

Figure S4: Fragmentation spectrum of synthesized AFB_1-*N*-acetylcysteine reference.

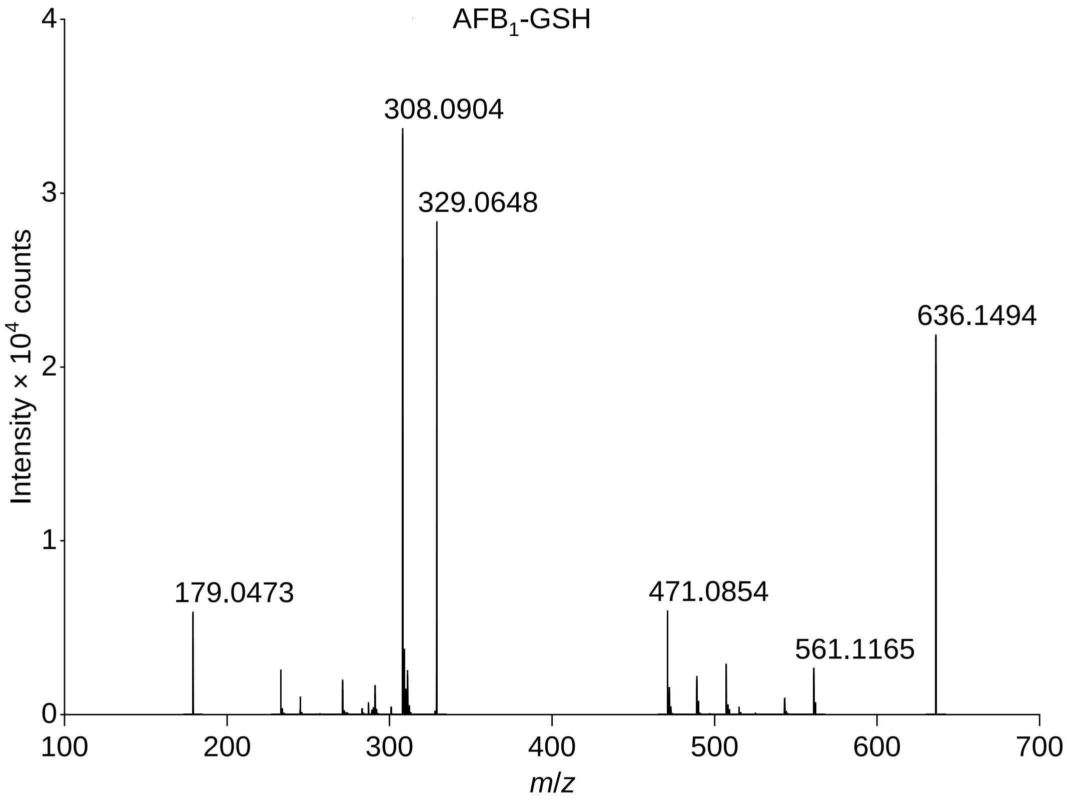

Figure S5: Fragmentation spectrum of synthesized AFB_1-GSH reference.

SPE clean-up of AFB_1-Lys in the samples

Table S11: SPE purification parameters for biological samples

SPE cartridge	Oasis MAX (1cc) (Waters)
**Conditioning**	1 mL MeOH
**Equilibration**	1 mL H_2O
**Washing**	1 mL H_2O
	1 mL MeOH/H_2O (7+3, v/v)
	1 mL 1 % ammonia in MeOH
	500 µL MeOH
**Elution**	1.5 mL 2 % FA in MeOH

HPLC-MS/MS methods for the metabolite quantification

Table S12: Analysis parameters for metabolite quantification.

**AFB_1 metabolites**				**AFB_1-Gua, 2´-deoxyguanosine**			
**Column**		Nucleodur Pyramid (150 × 2 mm, 3 µm), Agilent		**Column**		Nucleodur Pyramid (150 × 2 mm, 3 µm), Agilent	
**Detection**		MS/MS (Table S13)		**Detection**		MS/MS (Table S13)	
**Column**		40 °C		**Column**		40 °C	
**Autosampler**		7 °C		**Autosampler**		7 °C	
**Time [min]**	**Flow Rate [mL/min]**	**ACN + 0.1 % FA [%]**	**H_2O + 0.1 % FA [%]**	**Time [min]**	**Flow Rate [mL/min]**	**ACN + 0.1 % FA [%]**	**H_2O + 0.1 % FA [%]**
0	0.5	5	95	0	0.5	0	100
1	0.5	5	95	2	0.5	0	100
12	0.5	95	5	10	0.5	95	5
14.5	0.5	95	5	12	0.5	95	5
14.6	0.5	5	95	12.1	0.5	0	100
17	0.5	5	95	15	0.5	0	100

Table S13: Mass spectrometric parameters for metabolite quantification.

**Mass spectrometer**	QTRAP 6500 (AB Sciex)	
**ESI source**	Ion Drive, Turbo V Source	
**Source parameters**	Ion spray voltage	4500 V
	Source temperature	400 °C
	Curtain gas	35 psi
	Ion source gas 1	45 psi
	Ion source gas 2	55 psi
	Cell entrance potential	10 V
	Cell exit potential	11 V
	Ionisation mode	positive
**Scheduled MRM**	Analysis window	30 s
	Target scan time	0.2 s

Table S14: MRM transitions of quantified metabolites (QTRAP 6500).

Analyte	Precursor *m/z*	Product *m/z*	Adduct	CE	DP
**Aflatoxin B_1**	313.2	285.1	M+H	30	80
	313.3	241.0	M+H	50	80
**Aflatoxin M_1**	329.1	273.0	M+H	33	41
	329.1	229.0	M+H	53	41
**Aflatoxin P_1**	299.1	271.1	M+H	30	80
	299.1	215.1	M+H	38	80
**Aflatoxin Q_1**	328.7	206.0	M+H	33	80
	328.7	177.0	M+H	47	80
**Aflatoxicol**	297.0	269.1	$M-H_2O+H$	29	80
	297.0	115.0	$M-H_2O+H$	83	80
**AFB_1-GSH**	636.2	329.1	M+H	35	80
	636.2	308.1	M+H	35	80
**AFB_1-NAC**	492.1	329.1	M+H	20	80
	492.1	311.1	M+H	30	80
**AFB_1-Lys**	457.2	394.1	M+H	29	38
	457.2	328.1	M+H	43	38
**AFB_1-Gua**	480.1	329.1	M+H	25	80
	480.1	152.1	M+H	25	80
**AFB_1-FAPY**	498.1	329.1	M+H	20	80
	498.1	480.1	M+H	15	80
**2´-deoxyguanosine**	268.1	152.1	M+H	15	80
	268.1	135.0	M+H	50	80

HPLC-MS/MS method (AFB_1-lysine)

Table S15: Analysis parameters for metabolite quantification.

**Column**	Reprosil C18 AQ (150 × 2 mm, 3 µm), Dr. Maisch		
**Detection**	MS/MS (Table S16)		
**Column oven**	45 °C		
**Autosampler**	15 °C		
**Time [min]**	**Flow Rate [mL/min]**	**ACN + 0.1 % AA [%]**	**H_2O + 0.1 % AA [%]**
0	0.45	5	95
3	0.45	5	95
10	0.45	30	70
10.1	0.45	95	5
12	0.45	95	5
12.1	0.45	5	95
15	0.45	5	95

Table S16: Mass spectrometric parameters for quantification of AFB_1-lysine.

**Mass spectrometer**	QTRAP 7500 (AB Sciex)	
**Source**	OptiFlow Pro	
**Source parameters**	Ion spray voltage	2500 V
	Source temperature	550 °C
	Curtain gas	45 psi
	Ion source gas 1	65 psi
	Ion source gas 2	75 psi
	Ionisation mode	positive
	Scan type	MRM

Table S17: MRM transitions of quantified metabolites (QTRAP 7500).

	**Precursor *m/z***	**Product *m/z***	**Dwell time**	**EP**	**CE**	**CXP**
**AFB_1-Lys**	457.15	394.074	20	10	27	31
	457.15	410.958	10	10	15	5
	457.15	376.000	20	10	45	30
	457.15	328.000	10	10	42	30
**ISTD AFB_1-Lys**	465.15	400.100	10	10	27	31
	465.15	382.100	10	10	45	30
	465.15	329.100	10	10	42	30
	465.15	418.954	10	10	15	5

Quantification data

Table S18: Quantification data of metabolites in cell culture medium (matrix matched calibration with Williams E medium).

Metabolite	Conc.	Species	Time point	Dilution	Calibration range [ng/mL]	R^2
**AFB_1**	1 µM	Rat, Mouse, Human	10 min - 24 h	1+99	0.01-5	0.997
	10 µM	Rat, Mouse, Human	10 min - 24 h	1+99	0.25-25	0.999
**AFP_1**	1 µM	Rat, Human	10 min - 24 h	1+1	0.025-25	0.998
		Mouse	10 min - 6 h	1+99	0.01-10	0.998
			24 h	1+1	0.025-25	0.998
	10 µM	Rat, Human	10 min - 24 h	1+1	0.05-25	0.988
		Mouse	10 min - 24 h	1+99	0.25-10	0.998
**AFQ_1**	1 µM	Rat, Mouse, Human	10 min - 24 h	1+1	0.5-25	0.995
	10 µM	Rat, Mouse, Human	10 min - 24 h	1+1	0.5-25	0.995
**AFM_1**	1 µM	Rat, Mouse, Human	10 min - 24 h	1+1	0.025-25	0.999
	10 µM	Rat	10 min - 2 h	1+1	0.5-25	0.999
			4 h - 24 h	1+99	0.25-10	0.997
		Mouse	10 min - 24 h	1+1	0.5-25	0.999
		Human	10 min - 24 h	1+1	0.5-25	0.999
**AFL**	1 µM	Rat, Mouse, Human	10 min - 24 h	1+1	0.025-25	0.999
	10 µM	Rat, Mouse, Human	10 min - 24 h	1+1	0.025-25	0.999
**AFB_1-GSH**	1 µM	Rat, Mouse, Human	10 min - 24 h	1+1	0.1-25	0.997
	10 µM	Rat	10 min - 1 h	1+1	0.25-25	0.999
			2 h - 24 h	1+99	0.25-50	0.998
		Mouse	10 min	1+1	0.25-25	0.999
			30 min - 24 h	1+99	0.25-50	0.998
		Human	10 min - 24 h	1+1	0.25-25	0.999
**AFB_1-NAC**	1 µM	Rat, Mouse, Human	10 min - 24 h	1+1	0.025-25	0.998
	10 µM	Rat, Mouse	10 min - 6 h	1+1	0.025-25	1.000
			24 h	1+99	0.25-50	0.999
		Human	10 min - 24 h	1+1	0.025-25	1.000
**AFB_1-Lys**	1 µM	Rat, Mouse, Human	10 min - 24 h	1+1	0.025-25	0.997
	10 µM	Rat, Mouse, Human	10 min - 24 h	1+1	0.1-25	0.995
**AFB_1-Gua**	1 µM	Rat, Mouse, Human	10 min - 24 h	1+1	0.1-25	0.997
	10 µM	Rat, Mouse, Human	10 min - 24 h	1+1	0.25-25	0.998

Table S19: Quantification data of AFB_1-Gu and AFB_1-Lys in cell lysates after digestion (calibration in H_2O).

Metabolite	Sample	Species	Time point	Dilution	Calibration range [ng/mL]	R^2
**AFB_1-Gua**	1 µM, 10 µM	Rat, Mouse, Human	30 min, 2 h, 24 h	-	0.1-100	0.998
**dG**	1 µM, 10 µM	Rat, Mouse	30 min, 2 h, 24 h	1+99	5-1000	0.996
**AFB_1-Lys**	1 µM, 10 µM	Rat, Mouse, Human	30 min, 2 h, 24 h	SPE	0.005-5	0.998

Table S20:Quantification data of intracellular metabolites (calibration in H_2O).

Metabolite	Sample	Species	Time point	Dilution	Calibration range [ng/mL]	R^2
**AFB_1**	1 µM	Rat, Mouse	30 min, 2 h, 24 h	1+4	0.001-0.5	1.000
	10 µM	Rat, Mouse	24 h	1+4	0.001-0.5	1.000
	10 µM	Rat, Mouse,	30 min, 2 h	1+499	0.01-10	0.999
	1 µM	Human	30 min, 2 h, 24 h	1+499	0.01-10	0.999
	10 µM	Human	30 min, 2 h, 24 h	1+499	0.01-10	0.999
**AFP_1**	1 µM, 10 µM	Rat, Mouse, Human	30 min, 2 h, 24 h	1+4	0.0025-1	1.000
**AFQ_1**	1 µM, 10 µM	Rat, Mouse, Human	30 min, 2 h, 24 h	1+4	0.0025-0.5	0.999
**AFM_1**	1 µM, 10 µM	Rat, Mouse, Human	30 min, 2 h, 24 h	1+4	0.001-0.5	0.999
**AFL**	1 µM, 10 µM	Rat, Mouse, Human	30 min, 2 h, 24 h	1+4	0.001-1	1.000
**AFB_1-GSH**	1 µM, 10 µM	Rat, Mouse, Human	30 min, 2 h, 24 h	1+4	0.025-10	1.000
**AFB_1-NAC**	1 µM, 10 µM	Rat, Mouse, Human	30 min, 2 h, 24 h	1+4	0.0025-10	1.000
**AFB_1-Gua**	1 µM, 10 µM	Rat, Mouse, Human	30 min, 2 h, 24 h	1+4	0.01-10	1.000

The commercially available compounds (AFB_1, AFM_1, AFP_1, AFQ_1, AFL) were dissolved in ACN and the stock solutions were diluted with H_2O to a final concentration of 100 ng/mL. The AFB_1-adducts were stored in phosphate buffer and also diluted with H_2O to final concentrations of 100 ng/mL as stock solutions. The matrix-matched calibration was prepared from the stock solution adding the compounds and Williams E medium according to the dilution of the respective samples.

The quantification of AFB_1-Gua and dG in the DNA lysates was carried out without matrix in H_2O and the stock solutions used for this determination were dissolved also in H_2O at concentrations of 1 µg/mL AFB_1-Gua and 10 µg/mL dG.

Formation of AFP_1 glucuronide (AFP_1-Glc)

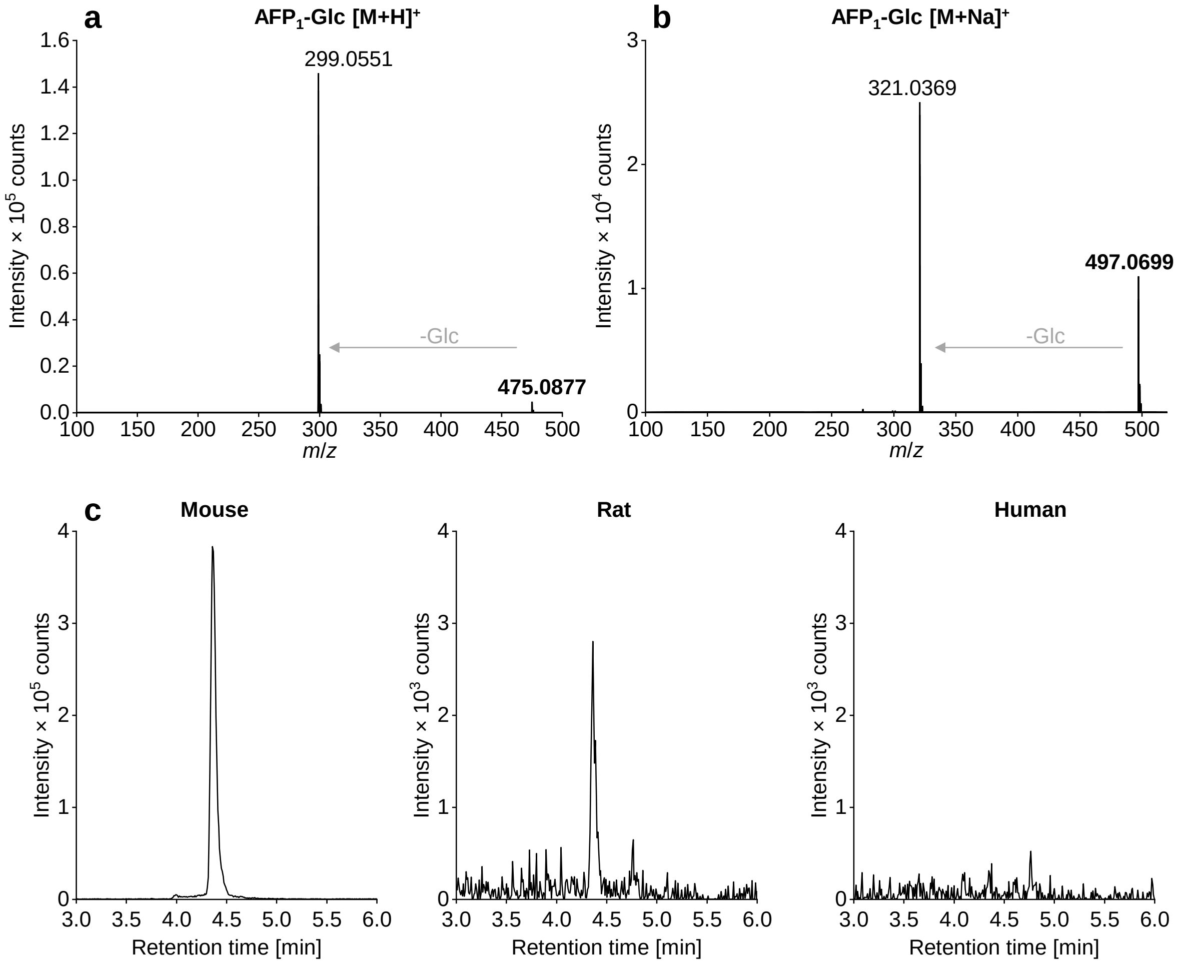

Figure S6: HPLC-HRMS fragmentation spectra of proton (**a**) and sodium adduct (**b**) of AFP_1-Glc with *m/z* 475.0877 and *m/z* 497.0699 showing a specific cleavage of glucuronide with AFP_1 as a product. The fragmentation spectra originated from the measurement of medium samples (24 h incubation of 10 µM AFB_1) from the mouse. Extracted ion chromatograms of *m/z* 475.0877 are shown to compare peak intensities of AFP_1-Glc in medium of mouse, rat and human hepatocytes after 24 h of incubation of 10 µM of AFB_1 (**c**). The medium was diluted 1 + 1 (*v*/*v*).

Formation rates of AFB_1 metabolites

Table S21: Formation rates of all metabolites calculated from medium samples.

		Formation rate [pmol/h]							
		**AFP_1**	**AFM_1**	**AFQ_1**	**AFL**	**AFB_1-GSH**	**AFB_1-NAC**	**AFB_1-Gua**	**AFB_1-Lys**
**1 µM (2 nmol)**	**Mouse**	475 471	15 069	15		17 864	574		89
	**Rat**	2019	8155	87	306	13 012	486		191
	**Human**	5	452	99	1 109				70
**10 µM (20 nmol)**	**Mouse**	1 208 204	248 906	46 686	1 163	296 311	1 440		3 376
	**Rat**	6426	62 581	74 700	6 702	64 529	1 858	657	1 673
	**Human**	46	3 649	7 116	18 923			39	903
